# Shrinkage vectors in flowable bulk-fill and conventional composites: bulk versus incremental application

**DOI:** 10.1007/s00784-020-03412-3

**Published:** 2020-07-11

**Authors:** Dalia Kaisarly, Moataz El Gezawi, Andreas Keßler, Peter Rösch, Karl-Heinz Kunzelmann

**Affiliations:** 1 Department of Conservative Dentistry and Periodontology, University Hospital, LMU Munich, Goethestrasse 70, 80336 Munich, Germany; 2 Biomaterials Department, Faculty of Oral and Dental Medicine, Cairo University, Cairo, Egypt; 3 Department of Restorative Dental Sciences, Imam Abdulrahman Bin Faisal University, Dammam, Saudi Arabia; 4 Faculty of Computer Science, University of Applied Sciences, Augsburg, Germany

**Keywords:** Flowable bulk-fill composite, Bulk application, Incremental application, Shrinkage vectors, Total-etch technique, Medical image registration

## Abstract

**Objectives:**

Sufficient depth of cure allows bulk-fill composites to be placed with a 4-mm thickness. This study investigated bulk versus incremental application methods by visualizing shrinkage vectors in flowable bulk-fill and conventional composites.

**Materials and methods:**

Cylindrical cavities (diameter = 6 mm, depth = 4 mm) were prepared in 24 teeth and then etched and bonded with OptiBond FL (Kerr, Italy). The composites were mixed with 2 wt% radiolucent glass beads.

In one group, smart dentin replacement (SDR, Dentsply) was applied in bulk “SDR-bulk” (*n* = 8). In two groups, SDR and Tetric EvoFlow (Ivoclar Vivadent) were applied in two 2-mm-thick increments: “SDR-incremental” and “EvoFlow-incremental.” Each material application was scanned with a micro-CT before and after light-curing (40 s, 1100 mW/cm^2^), and the shrinkage vectors were computed via image segmentation. Thereafter, linear polymerization shrinkage, shrinkage stress and gelation time were measured (*n* = 10).

**Results:**

The greatest shrinkage vectors were found in “SDR-bulk” and “SDR-increment2,” and the smallest were found in “SDR-increment1-covered” and “EvoFlow-increment1-covered.” Shrinkage away from and toward the cavity floor was greatest in “SDR-bulk” and “EvoFlow-increment2,” respectively. The mean values of the shrinkage vectors were significantly different between groups (one-way ANOVA, Tamhane’s T2 test, *p* < 0.05). The linear polymerization shrinkage and shrinkage stress were greatest in Tetric EvoFlow, and the gelation time was greatest in “SDR-bulk.”

**Conclusions:**

The bulk application method had greater values of shrinkage vectors and a higher debonding tendency at the cavity floor.

**Clinical relevance:**

Incremental application remains the gold standard of composite insertion.

## Introduction

The main drawback of resin-based composites is their shrinkage upon polymerization, leading to shrinkage stresses with possible debonding from the cavity walls, interfacial gap formation, and microleakage [1, 2]. Possible complications include discolored restoration margins, recurrent caries, undesirable pulp affections, and enamel cracks. Furthermore, the patient might complain of postoperative hypersensitivity [3–8]. The consequences of polymerization shrinkage are usually evaluated only with indirect methods, such as volumetric or linear shrinkage measurements outside a cavity [1, 2] or linear cuspal deflection measurements evaluated inside a cavity [9]. Other indirect measurements of the adverse effects of polymerization shrinkage are interfacial adaptation assessments at the margin of a restoration and bond strength tests [10–12]. Unfortunately, neither of these tests provides information on the real flow of material due to polymerization shrinkage. The nondestructive volumetric evaluation method of polymerization shrinkage using microcomputed tomography (micro-CT) scans displays areas of debonding and leakage around restorations but not internal displacement movements [13–20].

Bulk-fill composites were introduced with the intention of reducing application times due to thicker increments. The materials are optimized to ensure the higher increment thickness (<5 mm) is safely cured. The optimizations include photoinitiator systems that allow for greater depth of cure than conventional composites. Moreover, the shrinkage stresses are controlled via matrix and filler modifications. Smart dentin replacement (SDR) was the first clinically well-accepted bulk-fill material, and its volumetric polymerization shrinkage is lower than that of hybrid composites. Furthermore, the shrinkage stresses in SDR are lower than those in other bulk-fill composites [21, 22].

Similar to conventional composites, bulk-fill composites are available in various viscosities to fulfill the requirements of various application techniques. Flowable bulk-fill composites, such as SDR, are intended for use as a base below a layer of hybrid composite [23]. Bulk-fill composites with higher viscosity can be used as a direct posterior restorative material without the need for a covering hybrid composite [24]. The different viscosities are related to the filler content, which directly affects the elastic modulus. In the case of low-viscosity composites, stress reduction is achieved by a low elastic modulus, which allows the polymerization shrinkage to be compensated for by deformation of the restorative material [21].

The polymerization shrinkage and shrinkage stress can be evaluated in vitro with a simple sample geometry [2, 25, 26]. However, the real effects of polymerization shrinkage of a composite restoration can be seen only when applied in a cavity with its associated boundary conditions [27–32]. Earlier studies investigated shrinkage vectors of a flowable composite (Tetric EvoFlow, Ivoclar Vivadent, Schaan, Liechtensetin) applied with a 3-mm thickness; these studies were conducted before bulk-fill composites were widely available [30–33].

Bulk-fill composites have been optimized to enable higher increment thicknesses and to produce flowable bulk-fill composites with improved adaptation to the cavity walls. The aim of this study is to investigate—via shrinkage vector evaluation—the shrinkage behavior of SDR applied in 2-mm-thick and 4-mm-thick increments and compare the results to the polymerization shrinkage behavior of a conventional flowable composite, Tetric EvoFlow, applied in 2-mm-thick increments. Furthermore, the linear polymerization shrinkage, shrinkage stress, and gelation time of the composites are studied. The null hypothesis states that the application method—bulk versus incremental—does not influence the polymerization shrinkage behavior.

## Materials and methods

### Shrinkage vector evaluation

#### Sample preparation

A total of 24 intact human molars were gathered and deposited in sodium azide. The Ethics Committee of the Medical Faculty at the Ludwig-Maximilians—University of Munich, Germany approved the experimental procedures. The teeth were randomly divided into three groups according to the application method of the composite (*n* = 8). Cylindrical class I cavities (diameter = 6 mm; depth = 4 mm) were prepared in all samples, and the occlusal cusps of the teeth were flattened to ensure standardized perpendicular light-curing as close to the cavity as possible [30–33]. All restorations were bonded with the total-etch approach using OptiBond FL (Kerr, Scafati, Italy) and light-cured for 20 s at 1100 mW/cm^2^ (Bluephase Style, Ivoclar Vivadent, Schaan, Liechtenstein). Once per week, the light intensity was monitored with a dental radiometer (Bluephase Meter II, Ivoclar Vivadent, Schaan, Liechtenstein). The composites and the adhesive system used for the restorative procedures are listed in Table 1.

**Table 1:** Materials used in this study

Brand name	Composition	Batch no.	Manufacturer
SDR (flowable bulk-fill base)	SDR-patented urethane dimethacrylate resin Dimethacrylate resin Dysfunctional diluents barium and strontium alumino-fluoro-silicate glasses (68 wt%, 45 vol%) Photoinitiating system colorants	1,310,000,919	Dentsply, Konstanz, Germany
Tetric EvoFlow (nano-optimized flowable composite)	Bis-GMA, and urethane dimethacrylates (38 wt%) Barium glass filler, ytterbium trifluoride, highly dispersed silica, mixed oxide and prepolymers (62 wt%) Particle sizes of the inorganic fillers: 40–3000 nm	R36640	Ivoclar Vivadent, Schaan, Liechtenstein
Glass beads (radiolucent spheres, which are used as traceable markers)	SiO_2_ (72.50 wt%), Na_2_O (13.00 wt%), CaO (9.06 wt%), MgO (4.22 wt%), Al_2_O_3_ (0.58 wt%) Diameter: 40–70 μm	Art. no. 5211	Sigmund Lindner GmbH, Warmensteinach, Germany
OptiBond FL (prime/adhesive) (three-step total-etch adhesive)	Adhesive: hydroxyethylmethacrylate (HEMA) 15–20% Disodium hexafluorosilicate 1–2% Methacrylate ester monomers and inert fillers Primer: HEMA 25–30% Ethyl alcohol 20–25%	4,462,783 (prime) 4,462,763 (adhesive) “SDR-bulk” and “SDR-incremental” groups 5425092 (prime) 5430202 (adhesive) “EvoFlow-incremental” group	Kerr, Scafati, Italy
Gel etchant	37.5% phosphoric acid	4,466,220	Kerr, Scafati, Italy

In the first group, “SDR-bulk,” the bulk-fill material SDR (Dentsply, Konstanz, Germany) was placed in bulk (4-mm-thick increment), as suggested by the manufacturer [34], but not covered with a hybrid composite. In the second group, “SDR-incremental,” SDR was applied in two 2-mm-thick increments. In the third group, “EvoFlow-incremental,” the flowable composite Tetric EvoFlow (Ivoclar Vivadent, Schaan, Liechtenstein) was applied in two 2-mm-thick increments. Tetric EvoFlow was chosen as a representative material for low-viscosity composites and as a reference to earlier shrinkage vector evaluations [30–33].

#### Preparation of the traceable composite

Each flowable composite was mixed with silanized radiolucent glass beads (2 wt%), average particle size of 40–70 μm (Sigmund Lindner GmbH, Warmensteinach, Germany). The glass beads were silanized to adequately bond to the resin matrix [27, 35]. Tetric EvoFlow and SDR have, by coincidence, ideal radiopacity for the segmentation of glass beads. After the bonding procedure, the composite was applied to the prepared cavity and remained uncured during the first scan.

#### X-ray micro-CT measurements

The samples were scanned in a micro-CT apparatus (Micro-CT 40, Scanco Medical AG, Brüttisellen, Switzerland) at high resolution (8 μm voxel size) using an integration time of 300 ms. The settings for the micro-CT were a cathode current of 114 μA and an acceleration voltage of 70 kVp. To prevent dehydration and possible cracking of the tooth upon scanning, some drops of water were added to the sample holder in the space between the specimen and the cylinder of the sample holder without wetting the restoration. The sample holder was covered with a radiolucent dark cap during scanning to prevent any premature polymerization of the uncured composite.

**Fig. 1:**
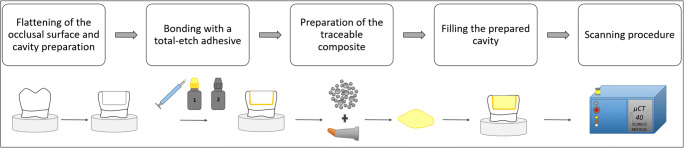
Workflow starting from the sample preparation to scanning the samples in the micro-CT

**Fig. 2:**
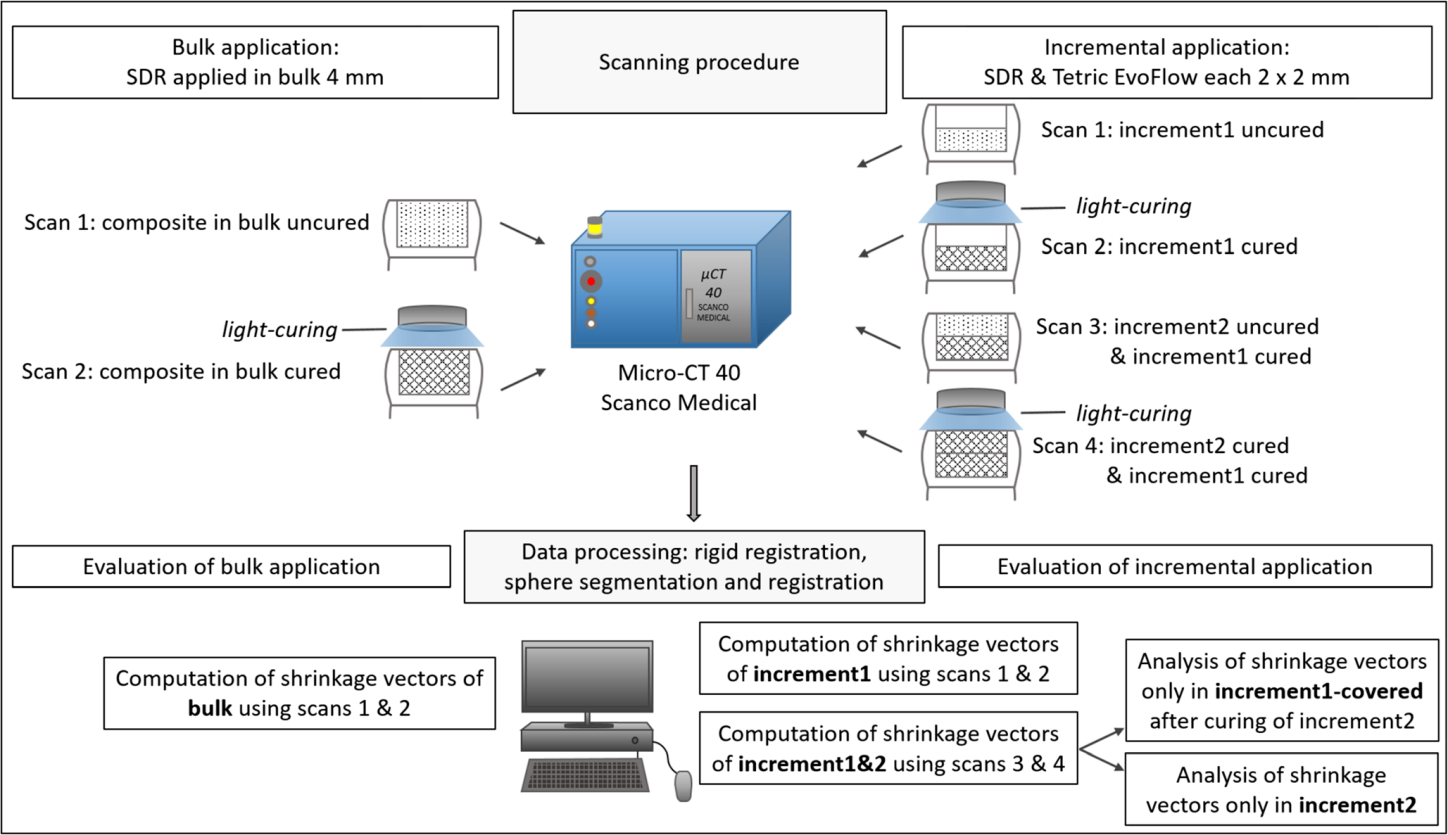
Scanning procedure and evaluation protocol. Details of the scanning procedure in the micro-CT for the composite applied in bulk or in two 2-mm-thick increments for obtaining the digital 3D data sets from the micro-CT. Data processing of the scans consisted of medical image analysis in the form of rigid registration and sphere segmentation and registration for computing the shrinkage vectors. In the bulk application, only two scans were evaluated. In the incremental application, four scans were evaluated. For the incremental application, the first increment was evaluated in the same manner as the bulk application, whereas the second increment was evaluated together with the underlying previously cured first increment because the first increment “increment1-covered” is influenced and deformed due to postpolymerization shrinkage and by the application of the second increment

In the bulk application and in the first increment of the incremental application, the sample with the uncured composite was placed inside the micro-CT machine for the first micro-CT scan (scan 1). Subsequently, the composite was light-cured for 40 s, and the sample was scanned with the cured composite (scan 2) using the same parameters. In the incremental application technique, each increment was light-cured for 40 s, and the restored tooth was scanned a total of four times; thus, the restoration was scanned with the second increment in the uncured state (scan 3) followed by light-curing and scanning the restoration with the second increment in the cured state (scan 4). The raw micro-CT scan data were reconstructed and saved; each scan (data set) was 5.2 GB in size. An overview of the workflow is displayed in Fig. 1, and details of the scanning procedure and the evaluation protocol are presented in Fig. 2.

#### Data processing

The first step of data processing consisted of rigid registration to perfectly overlap the pre- and postpolymerization scans via the outer tooth contours and the dentinoenamel junction. The second step consisted of sphere segmentation and sphere registration, in which each individual embedded glass bead was identified and located in both scans. Thus, the shrinkage vectors were calculated from the change in sphere position due to polymerization shrinkage in both scans. The methodology was previously described in detail [27, 31, 33].

The evaluation of the bulk application and “increment1” of the incremental application included scans 1 and 2. The evaluation of scans 3 and 4 provided results of the combined first and second increments represented as “SDR-increment1&2” and “EvoFlow-increment1&2.” The second increment was evaluated together with the underlying previously cured first increment because the first increment “increment1-covered” is influenced and deformed by the application of the second increment. Data from the two increments were separated according to the *z*-coordinate of the glass beads to display information on the shrinkage behavior of each respective increment separately: “increment1&2” was separated into “increment1-covered” and “increment2” (Fig. 2).

#### Visualization of the shrinkage vectors

The three-dimensional visualization of the shrinkage vector fields was performed using VTK (www.vtk.org), and each vector was represented graphically in the form of a glyph or arrow showing the direction of shrinkage. Thus, the shrinkage vector field was scaled by a factor of 5 to improve the visibility of the glyphs, and the shrinkage vector fields were analyzed qualitatively for shrinkage patterns [30–33].

#### Values of shrinkage vectors

The absolute values of the shrinkage vector magnitude were calculated from the change in position of each identified glass bead, defined by *x*-, *y*- and *z*-components, in both pre- and postpolymerization scans [30–33]. Furthermore, the axial movement of the glass beads along the cervico-occlusal axis was evaluated by examining only the *z*-component of the shrinkage vectors: negative values denote upward movement away from the floor toward the curing light, whereas positive values represent downward movement toward the cavity floor [30–32].

### Scanning electron microscopy

Examination of the internal adaptation was performed with one sample per group that was sectioned longitudinally and examined with a scanning electron microscope (ZEISS GEMINI® FESEM, SUPRA™ 55VP, Carl Zeiss SMT AG, Oberkochen, Germany) at × 200 magnification [30–32].

### Shrinkage stress and gelation time

The shrinkage stress of the investigated composites during polymerization was evaluated with a stress-strain analyzer (SSA T80, Engineering Consultancy Peter Dullin Jr., Munich, Germany) [36]. SDR was evaluated in a Teflon mold with dimensions of 4 mm × 4 mm × 4 mm to simulate the bulk application (C-factor = 0.5). For the incremental application, SDR and Tetric EvoFlow were applied to the Teflon mold with dimensions of 2 mm × 4 mm × 4 mm (C-factor = 0.33). Each composite application (*n* = 10) was light-cured for 20 s (Elipar Freelight2, 1200 mW/mm^2^), and the shrinkage force (N) was continuously recorded for 300 s and then divided over the area to obtain the shrinkage stress (MPa). The gelation time indirectly measures the time of stress accumulation resulting from network formation during polymerization. The gelation time is defined as the time until the shrinkage force exceeds the arbitrarily selected value of 0.5 N [37].

### Linear polymerization shrinkage

The linear polymerization shrinkage of SDR and Tetric EvoFlow was measured with the bonded disc method [1]. A fixed amount (2 g) of uncured composite (*n* = 10) was placed into the center of a ring on a glass plate, covered by a flexible microscope coverslip onto which a linear variable differential transformer (LVDT) probe was placed. Upon 20 s of light-curing (Elipar Freelight2, 1200 mW/mm^2^, 3 M ESPE, Seefeld, Germany), the deflection of the coverslip was measured, and the data were recorded with a computer for 300 s.

### Statistical analysis

Data were tested for normality using the Shapiro-Wilk test in IBM SPSS Statistics 22. The values of the shrinkage vectors, axial movement of glass beads, shrinkage stresses, and gelation time were subjected to a one-way analysis of variance (ANOVA) with post hoc pairwise comparisons using Tamhane’s T2 at *p* < 0.05, except for the gelation time, which was evaluated with Bonferroni’s post hoc comparison. The linear polymerization shrinkage percentage was tested using the independent *t* test at *p* < 0.05.

## Results

### Visualization of the shrinkage vectors

**Fig. 3:**
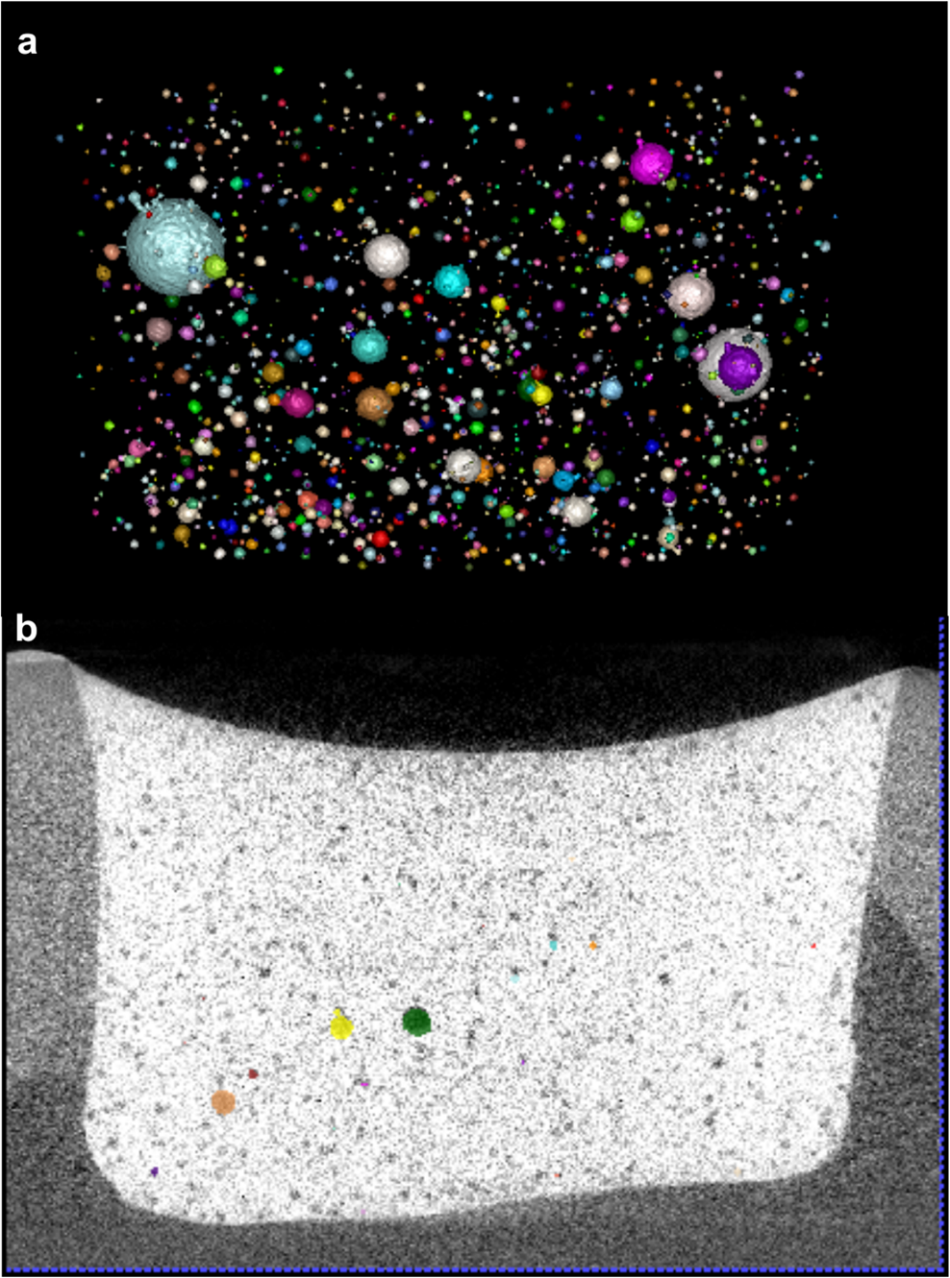
**a** Image segmentation through sphere segmentation identified the embedded glass beads as spheres, **b** and sphere registration overlapped the identified spheres in the prepolymerization and postpolymerization scans; colorless spheres belong to the prepolymerization scan, and colored spheres belong to the postpolymerization scan. The shrinkage vectors were computed from the displacement of the segmented spheres

The results of image segmentation through sphere segmentation and registration are shown in Fig. 3; the identified embedded glass beads were segmented and registered as spheres in the prepolymerization and postpolymerization scans. Then, the computation and visualization of the shrinkage vectors followed.

#### “SDR-bulk” group

**Fig. 4:**
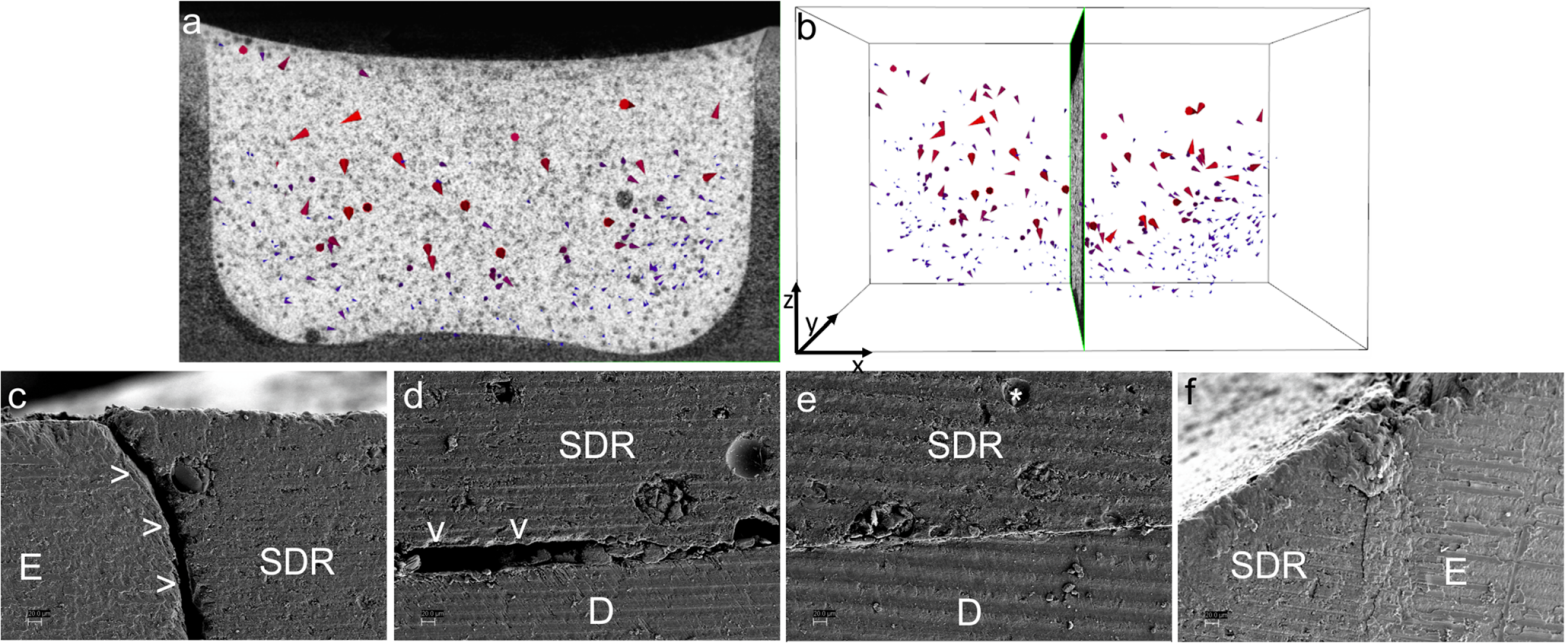
Shrinkage vector field of the “SDR-bulk” restoration with the **a** *x*-plane and **b** *y*-plane of the micro-CT scan in the background. Glyphs (or arrows) were scaled by a factor of 5 to enhance visibility. Small shrinkage vectors are seen at the lower restoration part pointing toward one side (right side) away from the location of the first debonding (left side). The SEM images (× 200 magnification) show **c** debonding at one margin (left) and **d** part of the cavity floor (arrows), whereas an intact bond was seen at **e** another part of the floor of the restoration and **f** the enamel margin (right). The star indicates a representative traceable glass bead

The upper part of the restoration in the “SDR-bulk” group showed large shrinkage vectors without a preferred direction. In the lower part of the restoration, many small shrinkage vectors pointed toward one side of the cavity (Fig. 4a–b). The scanning electron microscopy (SEM) images showed debonding at one side of the margin and part of the floor (Fig. 4c–d), whereas other locations had an intact bond (Fig. 4e–f).

#### “SDR-incremental” group

**Fig. 5:**
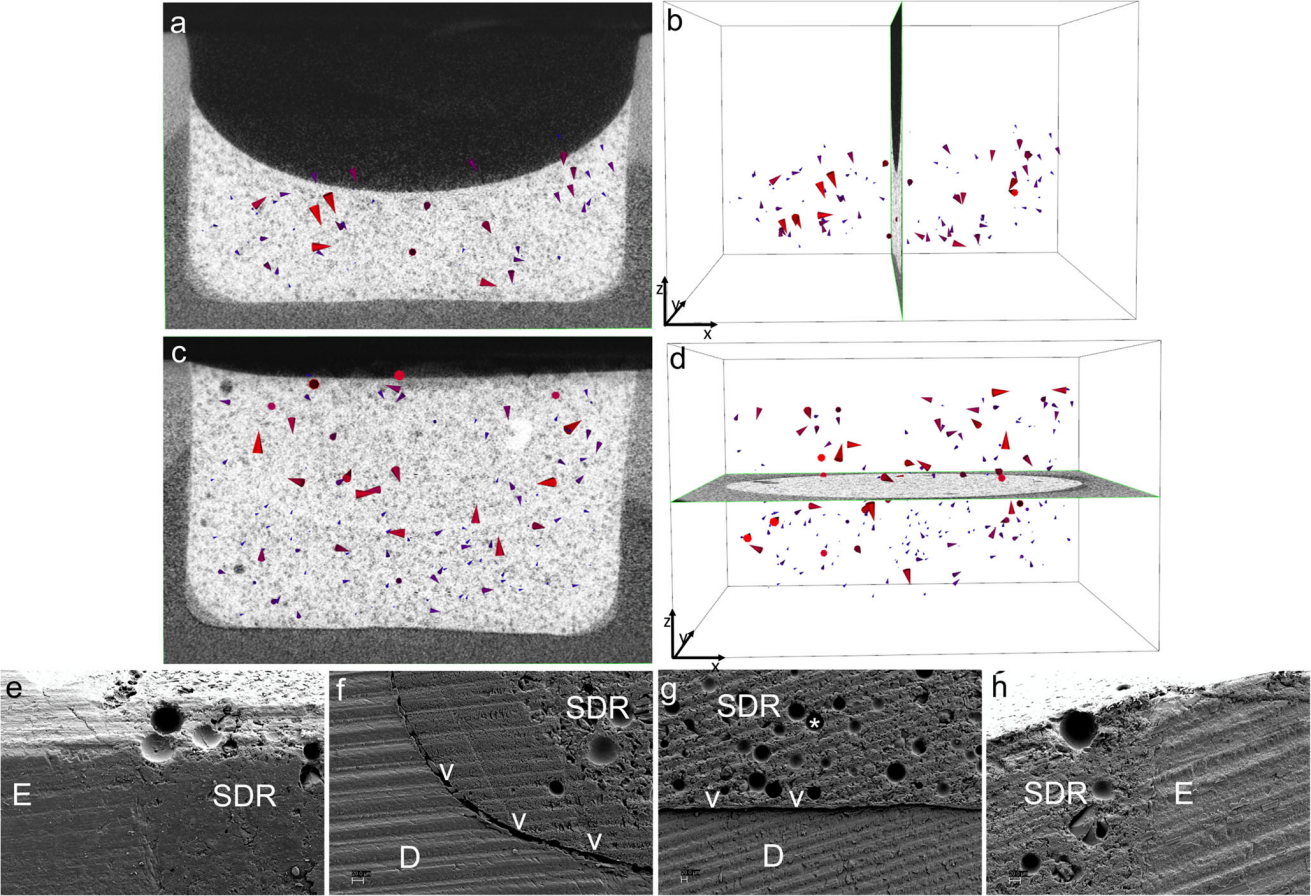
Shrinkage vector field of the “SDR-incremental” restoration with the **a**,**c** *x*-plane, **b** *y*-plane, and **d** *z*-plane of the micro-CT scan in the background. Glyphs (or arrows) were scaled by a factor of 5 to enhance visibility. “SDR-increment1” and “SDR-increment1&2” showed random movement of the shrinkage vectors with shrinkage vectors near the free surface showing downward movement. SEM images (× 200 magnification) display **e**,**h** intact bonds at both enamel margins and **f**,**g** debonding at the floor of the restoration (arrows). The star marks a traceable glass bead

In the upper part of the first increment “SDR-increment1,” the shrinkage vectors pointed downward toward the cavity floor, whereas the shrinkage vectors showed irregular arrangement in the lower part, in which only a few vectors pointed upward toward the curing light (Fig. 5a–b). In the second increment “SDR-increment2,” some shrinkage vectors pointed downward and some shrinkage vectors pointed sideways. The underlying first increment “SDR-increment1-covered,” which is the lower part of the whole restoration, displayed many small vectors pointing upward away from the cavity floor (Fig. 5c–d). The SEM images showed intact cavity margins and some debonding at the cavity floor but to a lesser degree than in the bulk application “SDR-bulk” (Fig. 5e–h).

#### “EvoFlow-incremental” group

**Fig. 6:**
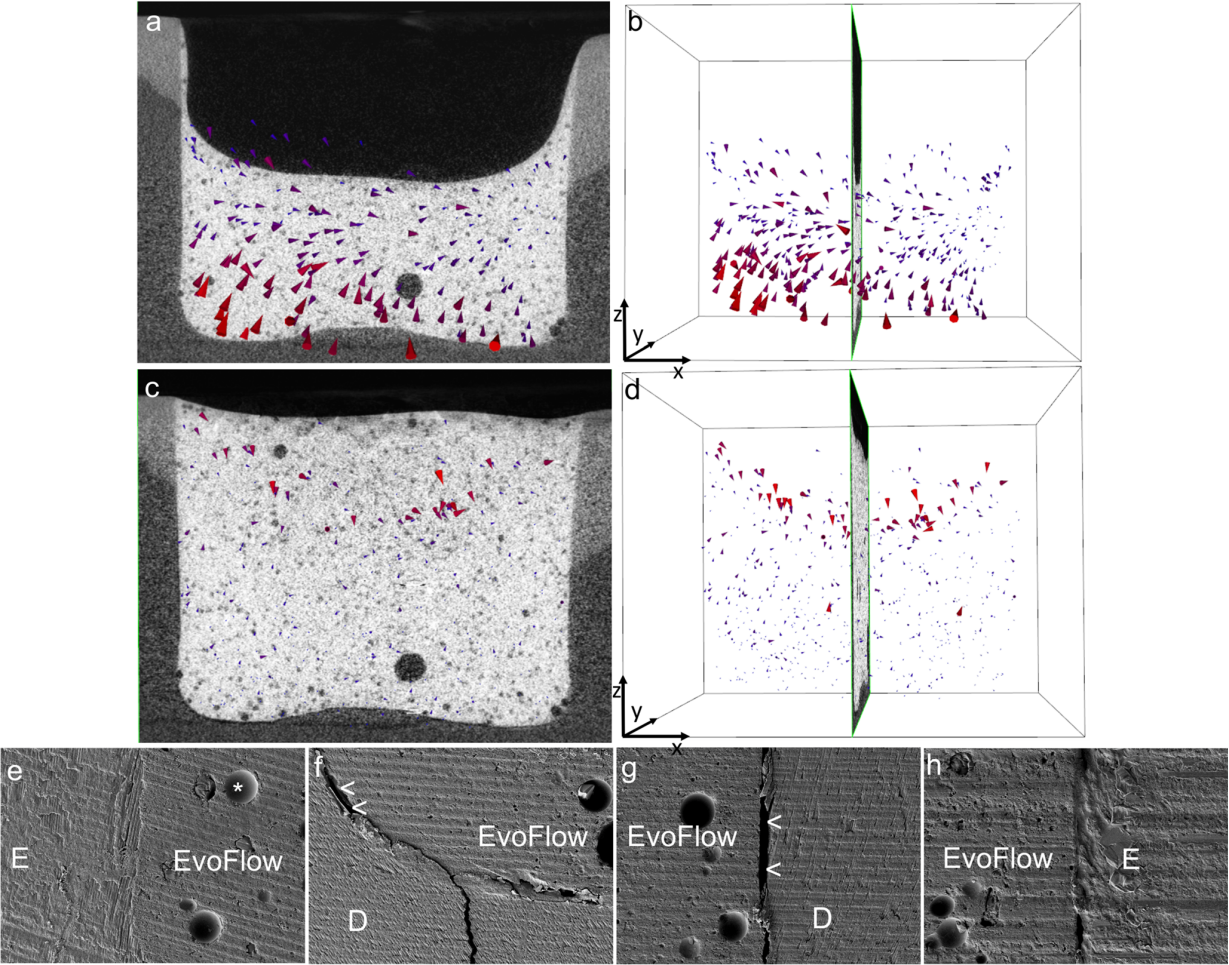
**a**, **b** Shrinkage vector field in the “EvoFlow-incremental” group, which showed large shrinkage vectors at the lower part of “EvoFlow-increment1” and small shrinkage vectors pointing downward at the free surface. **c**,**d** In “EvoFlow-increment1&2,” large shrinkage vectors pointed downward at the free surface of the restoration, whereas the remaining restoration displayed small shrinkage vectors with upward direction. SEM images (× 200 magnification) displayed an **e** intact bond on one side of the enamel margin, whereas the other side showed some areas of debonding **f** along the floor and **g**,**h** cavity walls (arrows). The star indicates a traceable embedded glass bead

In the first increment “EvoFlow-increment1,” the shrinkage vectors pointed upward and away from the cavity floor and then clearly deviated toward one side of the cavity. In the upper part of “EvoFlow-increment1,” small shrinkage vectors pointed downward, as shown in Fig. 6a–b. In the second increment “EvoFlow-increment2,” large shrinkage vectors pointed downward, whereas many very small vectors pointing away from the cavity floor were observed in the lower part of the restoration “EvoFlow-increment1-covered” (Fig. 6c–d). The SEM images displayed an intact bond on one side of the enamel margin (Fig. 6e), whereas debonding was observed in the other side of the enamel margin and along the cavity walls and the floor (Fig. 6f–h).

### Values of the shrinkage vectors

The data of the shrinkage vectors were not normally distributed (*p* < 0.05); however, one-way ANOVA requires only nearly normal data because it is robust to violations of normality and can still provide valid results, according to Winer et al. [38].

#### Quantitative nondirectional analysis

The largest values of shrinkage vectors were observed in groups “SDR-bulk” and “SDR-increment2.” In the incremental application method, the second increment of both composites showed greater values than the first increment, and the first increment below the second increment had the smallest values of shrinkage vectors in each composite. Table 2 shows the mean values of the shrinkage vectors, and one-way ANOVA revealed statistically significant differences between the groups (*F* = 1592.582; Df = 8, 47,745; *p* < 0.001) with post hoc Tamhane’s T2 test.

**Table 2:** Means and standard deviations of shrinkage vectors and glass bead movement in the *z*-direction (μm)

Group	Shrinkage vector length (μm) (SD)	Glass bead movement in the *z*-direction (μm) * (SD)
“SDR-bulk”	51.1 ± 37.8^a^	− 4.5 ± 36.2^a^
“SDR-increment1”	35.0 ± 27.0^b^	− 0.4 ± 23.0^b,c,d^
“SDR-increment1&2”	33.6 ± 27.9^b^	− 1.8 ± 21.4^b,c^
“SDR-increment1-covered”	22.1 ± 20.6^d^	− 3.7 ± 14.2^a^
“SDR-increment2”	49.6 ± 28.9^a^	0.8 ± 28.4^b,d^
“EvoFlow-increment1”	23.4 ± 14.7^d^	− 0.8 ± 19.0^b,c,d^
“EvoFlow-increment1&2”	20.8 ± 18.3^e^	5.7 ± 17.5^e^
“EvoFlow-increment1-covered”	12.6 ± 9.8^f^	− 0.3 ± 10.3^b,d^
“EvoFlow-increment2”	29.5 ± 21.1^c^	14.7 ± 20.2^f^

Different letters in one column denote a statistically significant difference between the groups, whereas the same letters show no significant difference between the groups.

*Negative values denote the upward movement of shrinkage vectors, whereas positive values represent the downward movement toward the cavity floor

#### Quantitative directional analysis

The greatest upward movement of the glass beads within the resin mass was detected in the bulk application method “SDR-bulk” and in the first increment of SDR “SDR-increment1-covered.” The greatest downward movement was seen in the second increment of Tetric EvoFlow “EvoFlow-increment2.” The mean values of the axial movement of glass beads are listed in Table 2, and one-way ANOVA revealed significant differences between the groups (*F* = 406.495; Df = 8, 47,127; *p* < 0.001) with post hoc Tamhane’s T2 test.

### Shrinkage stress and gelation time

The shrinkage stress was lower in the “SDR-bulk” group (0.97 ± 0.07 MPa) and in the “SDR-incremental” group (1.01 ± 0.06 MPa), and shrinkage stresses were significantly greater (*p* < 0.001) in the “EvoFlow-incremental” group (2.36 ± 0.46 MPa). The gelation time was significantly greater (*p* = 0.001) in the “SDR-incremental” group (4.03 ± 0.50 s) than in the “SDR-bulk” group (3.37 ± 0.50 s) and “Tetric EvoFlow-incremental” group (3.38 ± 0.46 s) (Table 3).

**Table 3:** Means and standard deviations of the shrinkage stress, gelation time and linear polymerization shrinkage percentage

Group		Shrinkage stress (MPa) (SD)	Gelation time (s) (SD)	Linear polymerization shrinkage (%) (SD)
SDR	Bulk 4 × 4 × 4 mm^3^	0.97 ± 0.07^a^	3.37 ± 0.50^a^	1.87 ± 0.08^a^
	Incremental 2 × 4 × 4 mm^3^	1.01 ± 0.06^a^	4.03 ± 0.28^b^	
Tetric EvoFlow	Incremental 2 × 4 × 4 mm^3^	2.36 ± 0.16^b^	3.38 ± 0.46^a^	2.35 ± 0.22^b^

Different letters in one column denote a statistically significant difference between the groups, whereas the same letters indicate no significant difference between the groups

### Linear polymerization shrinkage

The mean percentage of linear polymerization shrinkage according to the method of Watts and Cash [1] was significantly greater in Tetric EvoFlow (2.35 ± 0.22%) than in SDR (1.87 ± 0.08%) (Table 3).

## Discussion

The null hypothesis can be rejected because the polymerization shrinkage behavior of the applied flowable composites varied according to the application method. The shrinkage vector evaluation displayed greater mean values of shrinkage vectors in the bulk application than in the incremental application. However, the shrinkage stress and gelation time of SDR had smaller values in the bulk application.

In the pregel state, the composite mass movement induced by polymerization occurs due to flow, but postgel movement can occur due to strain within the polymerized composite and/or stress release after debonding from the cavity walls [39]. Pregel movement is influenced by the following factors: the radiant exposure of the curing light, which is influenced by the power output of the curing light, the distance to the composite, the direction of light application, and the focus of the curing light [40–43]; the value of the C-factor; the viscosity of the uncured composite; and the free shrinkage of the composite [4, 44, 45]. In our study, the C-factor was high and unfavorable where only the occlusal surface could shrink freely. Moreover, the viscosity was low in the flowable composites, which favors adaptation to the cavity walls.

Our analysis could visualize the basic components of mass movement upon polymerization of a composite by viewing the embedded glass beads, which serve as markers for tracing the mass movement in the polymerizing composite and for obtaining the values and the direction of the shrinkage vectors. The postgel mass movement due to polymerization is a result of shrinkage stress and is exaggerated in the case of the composite debonding from the cavity walls with an interfacial gap formation [39]. In this case, the shrinkage vectors are directed away from the interface or the cavity wall where debonding has occurred [31, 33].

The elastic deformation of a composite depends on the elastic modulus and the filler volume fraction [46]. Greater flexibility of the polymerizing SDR is achieved through the incorporation of patented modified urethane dimethacrylate and the use of a polymerization modulator, resulting in a slow polymerization rate that produces much less polymerization shrinkage stresses than those produced by conventional flowable composites [9, 47].

In general, the direction of the shrinkage vectors can be either isotropic or anisotropic: equal in all directions, a preference in one direction, which can be displayed by the shrinkage vectors in the *x*-, *y*- and *z*-directions, or in one single direction separately. In our study, we observed an anisotropic shrinkage pattern that could, among other things, be attributed to the curing light. The focus of the curing light was not homogenous throughout the tip of the light guide, and some minor movement occurred as the curing light was held by hand during the light-curing procedure [40, 41, 43]. Despite the shrinkage stress reduction, the greater values of the shrinkage vectors of SDR in the bulk application than in the incremental applications could be attributed to the greater volume of the restorative material [48].

The method of application of composites in our study had an influence on the magnitude and direction of the shrinkage vectors. Smaller shrinkage vectors in the first increment in each investigated composite can be attributed to the degree of proximity to the curing light of the successive increments and hence to the degree of conversion [41], which is in line with the total energy concept of light-curing composites [49].

The light beam profile of light-curing units is put in relation to the polymerization shrinkage and possible composite undercuring [40–43, 50–52]. Most light-curing tips deliver spots of high intensity, whereas other areas emit light of less intensity, which leaves the restorations unequally cured; thus, relying only on the output value might be insufficient. However, increasing the curing time improves the polymerization properties of bulk-fill composites [22]. Extended light-curing of 40 s in this study was performed in the shrinkage vector evaluation to overcome any variations in the light beam intensity [52]. However, the investigations into shrinkage stress, gelation time, and linear polymerization shrinkage were performed with 20-s light-curing as a separate independent test without performing any statistical correlation. The shrinkage stress and gelation time data of SDR have been published earlier and are reused with permission [53]. These data were added to the current study because they are beneficial for the understanding of the polymerization shrinkage behavior. This explains the difference in sample size between the shrinkage vector evaluation and the remaining tests.

The tooth-composite interface is the other influencing factor of the anisotropic shrinkage pattern resulting in debonding and gap formation due to compensatory mass movement through stress relaxation [48]. Although studying interfacial gaps on micro-CT scans was beyond the scope of the current research, some gaps were observed in the SEM images at the same site as the shrinkage vector origin. SEM was used as one representative sample of each study group for an adjunctive comparative evaluation. However, gap formation might occur upon sample sectioning and/or due to the effect of the high vacuum needed for SEM observation [54]. This constitutes a limitation of the study considering that the analysis was not performed on replicas and that a very good bonding system (Optibond FL) was used. Thus, a nondestructive evaluation of gaps using micro-CT scans would be advantageous [55].

Debonding of SDR from the cavity floor and/or margins was more pronounced when SDR was applied in bulk rather than when applied incrementally despite its low shrinkage stresses and flexibility [29, 56–58]. Debonding might be due to the inability of the developing interfacial bonds to completely counteract the developed shrinkage stresses, resulting in isolated areas of interfacial debonding at different locations [31]. Moreover, debonding from the cavity boundaries led to greater shrinkage vectors, as it allowed more freedom in shrinkage movement [31, 32]. In contrast to our findings, others have reported that bulk filling of SDR resulted in gap-free margins [56, 59] and higher fracture resistance of restorations [60, 61]. The total-etch adhesive was chosen for its high bond strength quality and reliable long-term performance [62–64].

The combination of the shrinkage vector evaluation, linear shrinkage measurement, shrinkage stress measurement, and gel point determination showed us a broader picture of the interaction of factors in bonded composite restorations. The lower shrinkage stress and the delayed gelation time of SDR enabled more material flow, which resulted in larger values of shrinkage vectors. However, the shorter gelation time of Tetric EvoFlow induced greater shrinkage stresses and shorter shrinkage vectors despite the greater linear shrinkage. The linear polymerization shrinkage measurement of a composite sample does not always reflect the shrinkage behavior of the composite when adhesively bonded to the cavity boundaries.

Variables that affect the values of the shrinkage vectors are overall shrinkage, degree of cure, bonding to the tooth structure, duration of the pregel phase, polymerization kinetics, and the elastic modulus of the composite [33, 43, 46, 65]. Upon comparing the values of the shrinkage vectors from the first increment of SDR and Tetric EvoFlow, we could detect larger values of shrinkage vectors in SDR; however, these values were smaller than those in the second increment. Even in the incremental application, SDR showed greater values of shrinkage vectors, although the volumetric shrinkage values of SDR (3.5 vol%) were lower than those of Tetric EvoFlow (4.2 vol%) [34, 66].

In the bulk application of SDR, the deviation in the shrinkage vectors toward one side was observed in the lower restoration part, which is in agreement with the findings reported by Chiang et al. [33]. The debonding could be attributed to weak bonding to dentin and/or stronger bonding to enamel on the opposite cavity wall [33, 44]. In our study, no variation in the thickness of the enamel margin was investigated or detected; thus, the shrinkage pattern can be attributed to the greater volume of composite than in the incremental application [29, 57, 58]. Others displayed shrinkage vectors that mainly indicated axial movement depending on the bonding condition, in which the unbonded composite moved upward toward the curing light, whereas the bonded composite exhibited shrinkage from the free surface downward toward the cavity floor, as seen in the second increment of our incremental groups [48, 65, 67].

In agreement with previous observations, the free surface of both investigated composites showed downward shrinkage, whereas earlier studies have applied the composite in bulk and not in increments [27, 65, 68]. The downward shrinkage of the free surface might be related to the strong bond of the second increment to the enamel margins. Moreover, the second increment was well bonded to the oxygen-inhibited layer of the first increment, which was pulled upward upon layering and was more pronounced in SDR. The lower degree of conversion of the first increment would apparently maintain greater flexibility in moving upwards, and the elastic moduli of SDR and Tetric EvoFlow are comparable (5.75 GPa and 5.1 GPa, respectively) [23, 24, 56]. Furthermore, the greater translucency of SDR than Tetric EvoFlow allows deeper penetration of the curing light, inducing greater values of shrinkage vectors that were visible in the upward movement of the previously cured first increment [69].

The dissimilar shrinkage behavior between the first and second increments could be related to the different bonding substrates [30]. In the first increment, the composite was bonded to the dentin cavity floor and walls, whereas the second increment was bonded to the composite underneath and to surrounding enamel margins and dentin cavity walls. Bonding to enamel yields higher bond strength values than bonding to dentin [28, 70], which influences the shrinkage vectors [33], thereby limiting the composite movement. Our results are in line with earlier findings that the boundary conditions in terms of bonding conditions and bonding substrates are the decisive factors governing the polymerization shrinkage direction [27, 31, 33, 44, 67].

Researchers have previously concluded that bulk-fill composites are not excellent substitutes for conventional composites [56, 71, 72] and that these materials developed lower bond strength values in large cavities than when applied incrementally [29]. The C-factor is still a critical deciding factor even in bulk-fill materials regarding the tooth-restoration interfacial integrity [73]. In addition to the filling technique, the composite type and size of the cavity affect the adhesion of the composite to the cavity floor [29, 57, 74].

The flowable bulk-fill composite exhibited smaller shrinkage vectors when applied incrementally. In large cavities, bulk filling reaches lower bond strength values than incremental application, and the cavity size is a determining factor affecting the bond strength [58, 74].

The current study reemphasizes the significance of micro-CT of composite restorations as a reliable nondestructive method of testing the resin composite shrinkage behavior at different regions within the bulk of the restoration as well as at the interfaces. The method of investigation employed in this study is highly accurate because it traces the actual movement of the embedded glass beads and allows the exact calculation of the three-dimensional shrinkage vectors [30–33]. This method provides insight into the internal shrinkage behavior in terms of shrinkage vectors relative to a specific location in the restored cavities, which allows more reliable prediction of the clinical performance of the individual composite. This advantage is lacking in other in vitro or in vivo investigations such as those involving only the volumetric investigation of polymerization shrinkage [2, 15, 27, 33, 44]. Thus, clinically relevant precise recommendations can be given to obtain a successful restoration.

Although SDR is intended to restore dentin and should be covered with a layer of hybrid composite [34], we limited our investigation to the effect of bulk versus incremental application. In deep cavities, such as the endodontic access cavity or deeply situated cavity margins of class II cavities, the bulk-fill composites are definitely of benefit due to their improved depth of cure. Clinicians are recommended to apply bulk-fill composites in increments to minimize the stresses on the bond, thereby improving the chances to preserve the tooth-restoration bond integrity.

## Conclusions

Under the circumstances of the current investigation, it could be concluded that the method of application influences the polymerization shrinkage behavior of composites. Bulk application of the bulk-fill composite SDR yields greater shrinkage vector values than incremental application. SDR shows random shrinkage patterns regardless of the insertion technique. Tetric EvoFlow produces a more regular shrinkage pattern than SDR. Debonding of composites in the incremental application is less likely to take place than in the bulk application.
